# Secure Multi-pArty Computation Grid LOgistic REgression (SMAC-GLORE)

**DOI:** 10.1186/s12911-016-0316-1

**Published:** 2016-07-25

**Authors:** Haoyi Shi, Chao Jiang, Wenrui Dai, Xiaoqian Jiang, Yuzhe Tang, Lucila Ohno-Machado, Shuang Wang

**Affiliations:** 1 Department of Biomedical Informatics, University of California, San Diego, CA 92093 USA; 2 Department of Electrical Engineering and Computer Science, Syracuse University, Syracuse, NY 13210 USA; 3 School of Electrical and Computer Engineering, University of Oklahoma, Tulsa, OK 74135 USA

## Abstract

**Background:**

In biomedical research, data sharing and information exchange are very important for improving quality of care, accelerating discovery, and promoting the meaningful secondary use of clinical data. A big concern in biomedical data sharing is the protection of patient privacy because inappropriate information leakage can put patient privacy at risk.

**Methods:**

In this study, we deployed a grid logistic regression framework based on Secure Multi-party Computation (SMAC-GLORE). Unlike our previous work in GLORE, SMAC-GLORE protects not only patient-level data, but also all the intermediary information exchanged during the model-learning phase.

**Results:**

The experimental results demonstrate the feasibility of secure distributed logistic regression across multiple institutions without sharing patient-level data.

**Conclusions:**

In this study, we developed a circuit-based SMAC-GLORE framework. The proposed framework provides a practical solution for secure distributed logistic regression model learning.

## Background

Biomedical research can benefit from data sharing from distributed sources. For example, comparative effectiveness research requires data comparison among different data sources to determine which existing health care interventions work best for certain patients. This requires a large amount of data to be harmonized. Big biomedical data networks, such as the patient-centered SCAlable National Network for Effectiveness Research (pSCANNER) clinical data research network (CDRN) [1], the Scalable Architecture for Federated Translational Inquiries Network (SAFTINet) [2] and the Electronic Medical Records and Genomics (eMERGE) Network [3] have been established to enable cross-institutional biomedical studies. However, information exchange of biomedical data (e.g., genome sequences, diagnoses, medication information, etc.) can put patient privacy at risk, where the potential risks include, but are not limited to, denial of certain types of insurance [4]. As a result, research participants may lose trust in research institutions, which may have an adverse impact on biomedical research. Privacy risks in biomedical studies have been demonstrated in many recent studies. For example, Vaidya demonstrated the possibility to re-identifying individuals from the public query system of the Healthcare Cost and Utilization Project (HCUP) [5]. Sweeney’s study successfully identified participants of the Personal Genome Project (PGP) [6]. In addition, with some background information, an attacker can even identify sensitive information of a participant using “anonymized” biomedical data [7–9]. The study in [10] demonstrated that aggregated genome statistics (e.g., allele frequencies) can present privacy risks. Therefore, it is imperative to develop privacy-preserving techniques to facilitate biomedical research. For this purpose, many distributed model learning frameworks [11–18] have been proposed for building a global model involving multiple participants, but without sharing sensitive patient-level information.

In this paper, we consider the scenario of horizontally partitioned data, where different institutions possess data from different patients, but with the same variables. Our previous work, Grid binary LOgistic REgression model (GLORE [11]) was developed to allow sharing models without necessarily sharing patient data in a distributed manner. It leveraged the aggregation of non-sensitive decomposable intermediary results to build a shared model. Its Bayesian extension, EXPLORER [19], proposed online learning to update the model with incremental data. It enabled asynchronous communication to alleviate probable service breakdown when coordinating multiple participants. Recently, grid ordinal and multinomial logistic regressions [13] were developed to deal with multi-center modeling with multiple categorical values for response variables. Remarkably, distributed modeling learning can also be employed in Cox regression for survival analysis [20–22].

Existing privacy-preserving solutions to multi-site regression [23, 24] can guarantee the precision of model learning. However, patient information could leak in these solutions due to disclosure of the information matrix and score vectors during iterative model learning [25, 26]. To protect these exchanged data, many secure multi-party computation (SMC) methods [18, 27–33] have been developed for distributed model learning. Unfortunately, existing SMC-based methods would still suffer from inappropriate disclosure under certain conditions due to the secure sum protocol. Therefore, El-Eman et al. [15] proposed the SPARK protocol that utilized different secure blocks to build a secure distributed logistic regression, which aims to offer stronger privacy protection for patient data. Although, homomorphic encryption based systems [34–36] can protect secure outsourcing, they need to assign the same public keys in the case of multi-party computation, which may leak intermediary results during communications among participants.

In this paper, we propose a secret-sharing circuit-based secure multi-party computation framework for grid logistic regression (SMAC-GLORE). Inheriting the distributed model learning framework from GLORE, SMAC-GLORE protects not only patient-level data, but also all the intermediary information exchanged during the model learning phase. Introducing secure multi-party computation to build boolean circuits for private data in learning, the proposed framework prevents participants from interpreting arbitrary intermediary information, such as aggregation of summary statistics, and only releases the final learned model parameters.

## Methods

To securely evaluate the logistic function, we introduced secret-sharing circuits-based Secure Multi-party Computation (SMC) into procedure of calculation. SMC provides a method for parties to jointly compute a function over their data while keeping the data private in semi-honest scenarios, where all the participants are those who are honest in running programs and algorithms correctly, but might be curious about the information transferred among entities.

### Garbled circuits

**Fig. 1:**
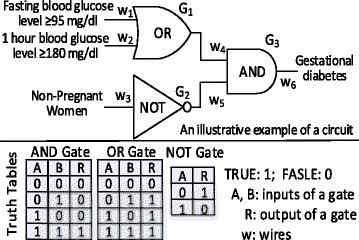
An example of a garbled circuit

The key idea of circuit based computation is based on the fact that operations in almost all modern digital computers are implemented by circuits combining basic logic gates such as AND, OR, NOT etc., where inputs and outputs of a gate may be TRUE or FALSE for certain propositions. One can design a garbled circuit counterpart [37–40] to protect the data and the computation. Figure 1 shows an example of diagnosing gestational diabetes based on blood glucose level (BGL) in a standard circuit representation, which consists of three gates (i.e., *G*_*i*_ with *i* = 1,2,3) and six wires (i.e., *w*_*j*_ with *j* = 1, 2,…, 6). Using Boolean algebra [41] and truth tables of the three basic gates shown in the figure, the circuit can calculate (Gestational diabetes) = (NOT (Non-Pregnant Women)) AND ((Fasting BGL ≥95 mg/dl) OR (1 h BGL ≥180 mg/dl)). In theory, one can build circuits of any complexity using basic logical gates to evaluate functions (e.g., secure distributed logarithm, exponent, etc.) or algorithms (e.g., secure distributed logistic regression models in this paper).

**Fig. 2:**
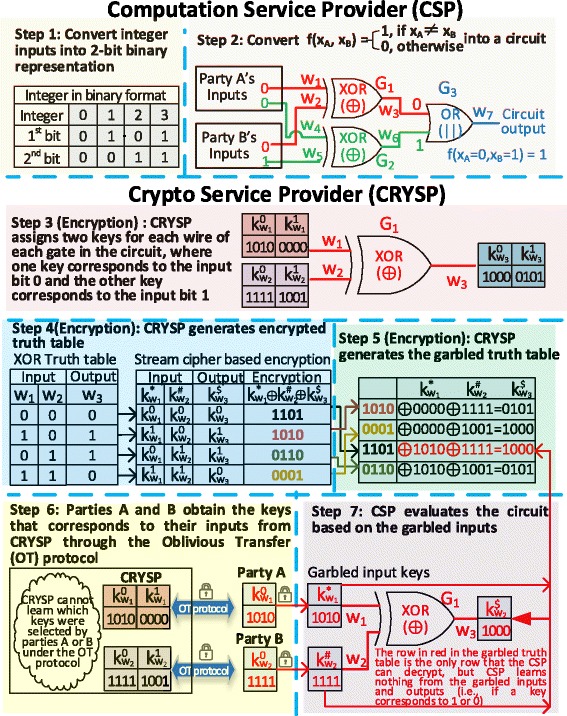
Private computations using garbled circuits, where parties A and B would like to compute whether two integers from them are identical, but without disclosing the actual integer value from each party

A garbled circuit [37, 39] is a specially designed circuit, which enables two (or more) parties to securely compute a function *f* (*x*_*A*_, *x*_*B*_) without exposing their private secrets (e.g., *x*_*A*_ and *x*_*B*_ are inputs from party A and party B, respectively). All parties here are assumed to be semi-honest collaborators, which means that they follow the protocol honestly, but may try to deduce additional information from the received messages during the protocols’ execution. Figure 2 illustrates several key steps to securely compare two integers using a garbled comparison circuit (GCC), where the comparison function can be formulated as $$ f\left({x}_A,\ {x}_B\right)=\left\{\begin{array}{c}\hfill 1,\  if\ {x}_A\ne {x}_B\hfill \\ {}\hfill 0,\  otherwise\hfill \end{array}\right. $$ with *x*_*A*_, *x*_*B*_ ∈ {0, 1, 2, 3}. The first step is to convert the integer inputs into a binary representation, where we use 2 bits to encode 4 different integers, i.e., {0 = 00; 1 = 01; 2 = 10, 3 = 11}. In the second step, a computation service provider (CSP) needs to build a circuit to implement the comparison function. The circuit consists of two XOR gates (i.e., G1 and G2) and performs bitwise comparisons between *x*_*A*_ and *x*_*B*_, where a XOR gate outputs 1 if its inputs are different, otherwise it outputs 0. The circuit utilizes an OR gate (i.e., G3) to compute whether any output of the two XOR gates is 1. For example, given inputs *x*_*A*_ *=* 0 and *x*_*B*_ *=* 1, the circuit outputs 1 in step 2 of Fig. 2 (i.e., *x*_*A≠*_*x*_*B*_). In the third step, a crypto service provider (CRYSP) generates two encryption keys for each wire of each gate in the circuit, where one key corresponds to bit 0 and the other to bit 1. Step 3 in Fig. 2 shows an example of assigning six keys for the XOR gate G1 with two input wires and one output wire. For the output wire w_3_, ${\mathrm{k}}_{{\mathrm{w}}_3}^0$ and ${\mathrm{k}}_{{\mathrm{w}}_3}^1$ are the randomly assigned keys (e.g., 1000 and 0101 in Fig. 2), which correspond to the wire outputs 0 and 1, respectively. Both keys were randomly chosen by the Crypto Service Provider (CRYSP) during the circuit initialization phase. In step 4, CRYSP will substitute the truth table of each gate with the encryption keys generated in step 3 and encrypt the outputs through a stream cipher based encryption scheme [42]. In step 5, CRYSP will generate a garbled truth table by randomly permuting rows of the encrypted truth table and sending it to the CSP for garbled circuit evaluation. In step 6, parties A and B will securely obtain the corresponding keys of their inputs (e.g., ${\mathrm{k}}_{\left({\mathrm{w}}_2\right)}^0=1111$ for input bit 0 of wire w_2_) from CRYSP through the oblivious transfer (OT) protocol [43], by which CRYSP cannot learn the actual selections of keys from the parties. In step 7, parties A and B will send their garbled keys (e.g., ${\mathrm{k}}_{{\mathrm{w}}_1}^{*}=1010$ and ${\mathrm{k}}_{{\mathrm{w}}_2}^{*}=1111$) to CSP, but without disclosing whether they refer to bit 1 or bit 0. Therefore, given the two garbled input keys, CSP can only decrypt one row (i.e., ${k}_{w_3}^{\$}=1000$) in the garbled truth table (as shown in red) without learning the underlying true value. Following the same protocol, CSP can evaluate the entire garbled circuit without learning any intermediary information. Finally, CSP sends the garbled key of the final output to CRYSP to find out whether it corresponds to 0 (unmatched) or 1 (matched). For garbled circuit based application, we can consider the circuit initialization phase as the encryption procedure, where encrypted truth table for each gate is created and distributed between two parties through the OT protocol.

In the case in which there is no trusted CRYSP, party A could, for example, serve as the CRYSP and party B could be CSP to avoid potential collusion risk between CSP and CRYSP. Moreover, one can choose more advanced encryption algorithms (other than the streaming cipher used in this example) to achieve a better protected OT protocol. The above example demonstrates how a secure integer comparison function can be achieved using garbled circuit-based SMC. In practice, advanced circuits are required to handle more complicated tasks, such as secure distributed logistic regression, where only the learned model parameters are allowed to be released as circuit outputs.

However, Yao’s garbled circuit is only secure in 2-party semi-honest scenarios, which is not sufficient for practical use. Usually, there are more than 2 parties or participants engaged in the same computing task. We based our model on the GMW project developed by Choi [44], who implemented the classical SMC protocols of Goldreich, Micali, and Wigderson (the GMW protocol) [45]. The GMW protocol uses secret-sharing rather than garbled truth tables to implement the secure computation, which enables the computation among more than 2 parties. In GMW, all the variables are represented as binary numbers and the protocol itself is able to protect against a semi-honest adversary with any number of corrupted parties. All the functions should be interpreted as boolean-circuits and each participant feeds the encrypted private data as input to the circuits. During the process of computing, none of the participants can interpret any temporary values except the final output. However, the GMW project only supports non-negative integers. We established our own encoding format, enabling the support for real number arithmetic, and built libraries for secure matrix operation primitives, which made it possible to solve the practical problems of building a secure distributed logistic regression model.

The proposed framework is based on the well-developed GMW protocol [44] for SMC. We overcame the limitations of GMW protocol and built several secure computation primitives to support SMAC-GLORE. As discussed in the original GMW implementation paper [44], the Naor-Pinkas OT was implemented as the encryption scheme to secure the computation. The mathematical definitions and proofs of security of the Naor-Pinkas OT protocol for GMW have been discussed in [46].

### Platform preparation

In our project, all the floating values are represented by binary vectors in 28-bits fixed-point format, in which 11 bits are assigned for the fractional part and the other 17 bits for the integer part. The highest bit of the integer part is reserved as a sign of positive or negative value. The two’s complement [47] method is adopted to represent negative values. Thus, all possible values under the proposed fixed-point format are ranging from − (2^28^ − 1) × 2^−11^ to (2^27^ − 1) × 2^−11^. The proposed platform extends the integer addition and multiplication to support floating number arithmetic. Here, we describe methods for implementing subtraction and multiplication.

#### Subtraction

When doing subtractions, we need to iteratively compare bits of minuend and subtrahend. If the bit in minuend is smaller than the corresponding bit in the subtrahend, we may need to borrow a bit leftwards. However, in circuits, it is very difficult and expensive to implement borrowing bits. Therefore, we choose to use two’s complement. To calculate the subtraction, we first calculate the two’s complement of the subtrahend, where we invert or flip all the bits of subtrahend and then add 1 to the least significant bit. After that, we add the calculated value to the minuend.

#### Multiplication

In multiplication, the result may, at most, double the number of required bits to represent the product. For example, the product of two *n*-bits numbers should have at most 2*n* bits. However, the GMW project requires the two factors involved in a multiplication to have the same number of bits, and allocates the same number of bits for the product. So, if we still use 28 bits for multiplication, we will suffer from significant precision loss or computing error. To solve this problem, we double the size of all the values when doing multiplication. In another word, we expand the 28-bits values to 56-bits values before multiplication and use the 56-bits values for the multiplication. As the calculated result will also have 56 bits, we need to drop 28 bits. Even though, in this procedure, we may waste a lot of bits and computing effort, we can secure the computing precision.

Based on the basic operations described above, we built several secure primitives in the GMW project for matrix operations, including matrix addition, matrix subtraction and matrix multiplication, which are elaborated in detail later in this section.

### Problem definition

For clarity, in the remainder of this paper, we reserve regular symbols to scalar variables and bold symbols to vectors or matrices. Logistic regression is widely used in biomedical decision support applications, including feature selection, survival analysis, etc. Suppose there is a training dataset $\mathcal{D}=\left\{\left(\boldsymbol{X},\boldsymbol{y}\right)\right\}=\left\{\left({\boldsymbol{x}}_1,{y}_1\right),\cdots \left({\boldsymbol{x}}_n,{y}_n\right)\right\}$ of *n* records for patients, where *y*_*i*_ ∈ {0, 1} is the observed binary outcome and ***x***_*i*_ corresponds to the *m*-dimensional covariates for the *i*-th patient. In the logistic regression model, the likelihood function of *y*_*i*_ *=* 1 given ***x***_*i*_ can be expressed as follows,

1 $$ P\left({y}_i=1\Big|{\boldsymbol{x}}_i,\boldsymbol{\beta} \right) = \frac{1}{1+{e}^{-{\boldsymbol{\beta}}^T{\boldsymbol{x}}_i}}, $$

where ***β*** is a parameter vector that measures the relationship between the response variable *y*_*i*_ and covariates ***x****i*. Note that *P*(*y*_*i*_ = 0|***x***_*i*_, ***β***) = 1 − *P*(*y*_*i*_ = 1|***x***_*i*_, ***β***) for binary response variable *y*_*i*_. Given the training dataset $\mathcal{D}$, ***β*** can be estimated through maximization of the following log likelihood function

2 $$ \widehat{\boldsymbol{\beta}}={\mathrm{argmax}}_{\boldsymbol{\beta}}\left(l\left(\boldsymbol{\beta} \right)=-{\displaystyle {\sum}_{i=1}^n \log \left(1+{e}^{-{\boldsymbol{\beta}}^T{\boldsymbol{x}}_i}\right)}\right). $$

Here, we use *l* (***β***) to represent the log-likelihood function. Since there is no closed-form solution for ***β***, iterative numerical solutions are required to obtain the optimal parameters. In a centralized model, the Newton-Raphson method is widely used to find $\widehat{\boldsymbol{\beta}}$. The iterative maximization is achieved by calculating the first and second derivatives of the log-likelihood function *l*(***β***). In the *t*-th iteration, current estimation ***β***^(*t*)^ is updated by
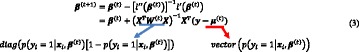


In Eq. (3), ***W***^(*t*)^ is the diagonal matrix with elements *p*(*y*_*i*_ = 1|***x***_*i*_, ***β***^(*t*)^)*p*(*y*_*i*_ = 0|***x***_*i*_, ***β***^(*t*)^) and ***μ***^(*t*)^ is the vector of probabilities *p*(*y*_*i*_ = 1|***x***_*i*_, ***β***^(*t*)^). Since the Hessian matrix ***H*** = ***X***^***T***^***W***^(*t*)^***X*** is a square matrix of second partial derivatives *l*^' '^(***β***), the iterative procedure can be rewritten as

4 $$ {\boldsymbol{\beta}}^{\left(t+1\right)}={\boldsymbol{\beta}}^{(t)}-{\boldsymbol{H}}^{-1}\ {l}^{\hbox{'}}\left(\ {\boldsymbol{\beta}}^{(t)}\right). $$

In this study, we consider a distributed model learning problem, where $\mathcal{D}$ is horizontally partitioned by *h* parties as $\mathcal{D}=\left\{\left({X}^1,{y}^1\right),\cdots, \left({X}^h,{y}^h\right)\right\}$. For the *j*-th party *p*_*j*_, ${\boldsymbol{X}}^j=\left({\boldsymbol{x}}_1^j\ {\boldsymbol{x}}_2^j\cdots {\boldsymbol{x}}_{n_j}^j\right)$ is a *m* × *n*_*j*_ matrix representing the subset of *n*_*j*_ covariates from *p*_*j*_, and ${\boldsymbol{y}}^{\boldsymbol{j}}=\left({y}_1^j,{y}_2^j,\cdots, {y}_{n_j}^j\right)$ is the vector of *n*_*j*_ corresponding binary response variables. Consequently, the intermediary results for the logistic regression model in Eq. (3) can be linearly decomposed [11] as

5 $$ \begin{array}{l}{\boldsymbol{X}}^T{\boldsymbol{W}}^{(t)}\boldsymbol{X}={\displaystyle \sum_{j=1}^h}{\left({\boldsymbol{X}}^j\right)}^T{\left({\boldsymbol{W}}^j\right)}^{(t)}{\boldsymbol{X}}^j\\ {}{\boldsymbol{X}}^T\left(\boldsymbol{y}-{\boldsymbol{\mu}}^{(t)}\right)={\displaystyle \sum_{j=1}^h}{\left({\boldsymbol{X}}^j\right)}^T\left({\boldsymbol{y}}^j-{\left({\boldsymbol{\mu}}^j\right)}^{(t)}\right)\end{array} $$

Equation (5) shows that each party can calculate its own intermediary results conditioned on its local data (***X***^*j*^, ***y***^*j*^), and share them for the combined results. However, this method requires a trusted server [11] to exchange local statistics. In this paper, we will build a decentralized framework for logistic regression using secret-sharing circuits based on secure multi-party communication. The proposed framework protects the intermediary statistics (***X***^*j*^)^*T*^(***W***^*j*^)^(*t*)^***X***^*j*^ and (***X***^*j*^)^*T*^(***y***^*j*^ − (***μ***^*j*^)^(*t*)^) with a joint function for all the parties without disclosing any private information.

Algorithm 1 (A1) summarizes the key steps in the proposed SMAC-GLORE framework. Each participant provides encrypted data ***X***^*j*^ as input. The only output of the algorithm is the learned model coefficients ***β***. All the intermediary information exchange is protected by the OT protocol and secret-sharing circuits. In A1: line 1, each party can locally calculate its own part of the fixed-Hessian matrix ${\tilde{H}}_i$ (see Eq. (7)) and feed it as part of input to the circuit, while within the circuit, the fixed-Hessian matrix is securely constructed as $\tilde{\boldsymbol{H}}={\displaystyle {\sum}_{i=1}^h{\tilde{H}}_i}$ based on Eq. (7). In A1 line 2, we apply the proposed secure matrix inversion algorithm (see Secure Hessian matrix inversion section) to get the inversion of $\tilde{\boldsymbol{H}}$, where we implement the Strassen Algorithm to speed up the matrix multiplication and improve performance. Then we iteratively update ***β*** until it converges via A1 lines 4–7, where we first securely construct ***X*** from each participant’s input and repeat the procedure described in Eq. (4). Within each iteration, we update the first derivative of the maximum likelihood function with the current ***β*** (A1: line 5). In this procedure, reciprocals or divisions are required and we use the same procedure as for matrix inversion.
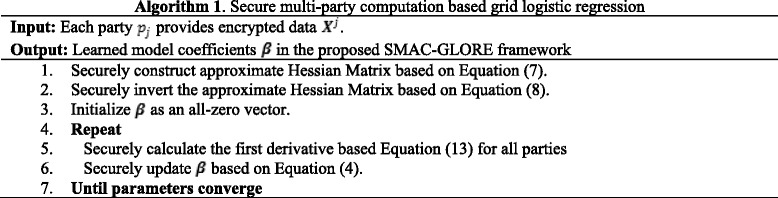


### Secure Hessian matrix inversion

In this study, the distributed Newton-Raphson method [11, 12] is adopted to compute the coefficients ***β*** across multiple parties. Since the Hessian matrix ***H*** represents the matrix of the second partial derivatives of maximum likelihood function *l*(***β***), it has to be updated with the most up-to-date ***β***^(***t***)^ and inverted for each iteration. However, calculating the inversion of ***H*** would significantly increase the computational burden and could be unfeasible for our secret-sharing, circuit-based method. One workaround to reduce the complexity is to replace the varying Hessian Matrix with a fixed matrix. According to Böhning [48], the true Hessian matrix can be approximated by

6 $$ \boldsymbol{H}=-\frac{1}{4}{\boldsymbol{X}}^T\boldsymbol{X} $$

where ***X*** = [***X***^1^ ***X***^2^ ⋯ ***X***^*h*^]. Consequently, the approximated Hessian Matrix can be rewritten as

7 $$ \tilde{\boldsymbol{H}}=-\frac{1}{4}{\displaystyle \sum_{j=1}^h}{\left({\boldsymbol{X}}^j\right)}^T{\boldsymbol{X}}^j $$

Equation (7) implies that each party *p*_*j*_ can locally calculate its own part of a partial Hessian matrix ${\tilde{\boldsymbol{H}}}_i=-{\left({X}^j\right)}^T{X}^j/4$, and feed ${\tilde{\boldsymbol{H}}}_j$ as part of the input to the circuit. While in the circuit, the approximated Hessian matrix is constructed by aggregating all the partial Hessian matrices as $\tilde{\boldsymbol{H}}={\displaystyle {\sum}_{j=1}^h{\tilde{\boldsymbol{H}}}_j}$.

To further reduce the computational complexity of inverting $\tilde{\boldsymbol{H}}$, we use the approximating method introduced by Nardi [49]. The numerical approximation method iteratively computes ${\tilde{\boldsymbol{H}}}^{-1}$ according to Eq. (8).

8 $$ \begin{array}{l}{\boldsymbol{N}}_{t+1}=2{\boldsymbol{N}}_t-{\boldsymbol{N}}_t{\boldsymbol{M}}_t,\kern1.25em {\boldsymbol{N}}_0={c}^{-1}\boldsymbol{I},\\ {}{\boldsymbol{M}}_{t+1}=2{\boldsymbol{M}}_t-{\boldsymbol{M}}_t^2,\kern2em {\boldsymbol{M}}_0={c}^{-1}\tilde{\boldsymbol{H}},\end{array} $$

where ${\boldsymbol{M}}_t={\boldsymbol{N}}_t\ \tilde{\boldsymbol{H}}$, and *c* is constant. After convergence (e.g., in ~10 to 15 iterations), ***N***_*t*_ will provide an accurate approximation to the inversion of $\tilde{\boldsymbol{H}}$.

### Matrix multiplication

We transfer the matrix inversion problem into an iterative procedure of matrix multiplication and addition. Therefore, optimizing the implementation of matrix multiplication can improve the efficiency of the proposed framework. In this subsection, we adopted the Strassen algorithm for matrix multiplication.

Let us denote ***A*** and ***B*** two square *n* × *n* matrices and *C* = ***AB*** their matrix product. Here, *n* is recommended to be a power of 2, namely *n* = 2^*k*^, *k* ∈ ℕ. ***A****,****B*** and *C* can be partitioned into equally sized block matrices as

9 $$ \boldsymbol{A}=\left[\begin{array}{cc}\hfill {\boldsymbol{A}}_{1,1}\hfill & \hfill {\boldsymbol{A}}_{1,2}\hfill \\ {}\hfill {\boldsymbol{A}}_{2,1}\hfill & \hfill {\boldsymbol{A}}_{2,2}\hfill \end{array}\right],\ \boldsymbol{B} = \left[\begin{array}{cc}\hfill {\boldsymbol{B}}_{1,1}\hfill & \hfill {\boldsymbol{B}}_{1,2}\hfill \\ {}\hfill {\boldsymbol{B}}_{2,1}\hfill & \hfill {\boldsymbol{B}}_{2,2}\hfill \end{array}\right],\ \boldsymbol{C} = \left[\begin{array}{cc}\hfill {\boldsymbol{C}}_{1,1}\hfill & \hfill {\boldsymbol{C}}_{1,2}\hfill \\ {}\hfill {\boldsymbol{C}}_{2,1}\hfill & \hfill {\boldsymbol{C}}_{2,2}\hfill \end{array}\right], $$

where ***A***_*i,j*_, ***B***_*i,j,*_ and ***C***_*i,j,*_ are all (*n*/2) × (*n*/2) matrices. According to the definition of block matrix multiplication, ***C***_*i,j,*_ can be represented by ***A***_*i,j,*_ and ***B***_*i,j,*_ for *i*, *j* = 1, 2.

10 $$ \begin{array}{l}{\boldsymbol{C}}_{1,1}={\boldsymbol{A}}_{1,1}{\boldsymbol{B}}_{1,1}+{\boldsymbol{A}}_{1,2}{\boldsymbol{B}}_{2,1},\kern0.75em {\boldsymbol{C}}_{1,2}={\boldsymbol{A}}_{1,1}{\boldsymbol{B}}_{1,2}+{\boldsymbol{A}}_{1,2}{\boldsymbol{B}}_{2,2},\\ {}{\boldsymbol{C}}_{2,1}={\boldsymbol{A}}_{2,1}{\boldsymbol{B}}_{1,1}+{\boldsymbol{A}}_{2,2}{\boldsymbol{B}}_{2,1},\kern0.75em {\boldsymbol{C}}_{2,2}={\boldsymbol{A}}_{2,1}{\boldsymbol{B}}_{1,2}+{\boldsymbol{A}}_{2,2}{\boldsymbol{B}}_{2,2}.\end{array} $$

According to Eq. (10), the calculation of ***C*** requires the same number of multiplications as the standard definition of matrix multiplication ***C*** = ***AB***. To reduce the number of multiplications, we introduce the Strassen algorithm which defines some new matrices based on ***A***_*i,j,*_ and ***B***_*i,j,*_.

11 $$ \begin{array}{l}{\boldsymbol{M}}_1={\boldsymbol{A}}_{1,1}\left({\boldsymbol{B}}_{1,2}-{\boldsymbol{B}}_{2,2}\right),\kern0.75em {\boldsymbol{M}}_2=\left({\boldsymbol{A}}_{1,1}+{\boldsymbol{A}}_{1,2}\right){\boldsymbol{B}}_{2,2},\ \\ {}{\boldsymbol{M}}_3=\left({\boldsymbol{A}}_{2,1}+{\boldsymbol{A}}_{2,2}\right){\boldsymbol{B}}_{1,1},\kern0.75em {\boldsymbol{M}}_4={\boldsymbol{A}}_{2,2}\left(\ {\boldsymbol{B}}_{2,1}-{\boldsymbol{B}}_{1,1}\right),\\ {}{\boldsymbol{M}}_5 = \left({\boldsymbol{A}}_{1,1}+{\boldsymbol{A}}_{2,2}\right)\left(\ {\boldsymbol{B}}_{1,1}+{\boldsymbol{B}}_{2,2}\right),\kern0.75em {\boldsymbol{M}}_6 = \left({\boldsymbol{A}}_{1,2}-{\boldsymbol{A}}_{2,2}\right)\left(\ {\boldsymbol{B}}_{2,1}+{\boldsymbol{B}}_{2,2}\right),\\ {}{\boldsymbol{M}}_7=\left({\boldsymbol{A}}_{1,1}-{\boldsymbol{A}}_{2,1}\right)\left(\ {\boldsymbol{B}}_{1,1}+{\boldsymbol{B}}_{1,2}\right)\end{array} $$

Equation (11) requires only 7 matrix multiplications between (*n*/2) × (*n*/2) square matrices (one for each ***M***_*l*_, *l* = 1, ⋯, 7) to calculate ***C*** = ***AB***, which reduces the number of multiplications by *n*^3^/8. The product *C* can be recovered from ***M***_*l*_, *l* = 1, ⋯, 7 by

12 $$ \begin{array}{l}{\boldsymbol{C}}_{1,1}={\boldsymbol{M}}_5+{\boldsymbol{M}}_4-{\boldsymbol{M}}_2+{\boldsymbol{M}}_6,\kern0.75em {\boldsymbol{C}}_{1,2}={\boldsymbol{M}}_1+{\boldsymbol{M}}_2,\\ {}{\boldsymbol{C}}_{2,1}={\boldsymbol{M}}_3+{\boldsymbol{M}}_4,\kern0.75em {\boldsymbol{C}}_{2,2}={\boldsymbol{M}}_5+{\boldsymbol{M}}_1-{\boldsymbol{M}}_3-{\boldsymbol{M}}_7.\end{array} $$

The matrices can be iteratively partitioned *k* times, when *n* = 2^*k*^. Thus, the Strassen algorithm can reduce the complexity of matrix multiplication from *O*(*n*^3^) to *O*(*n*^2.8^).

In this work, the Strassen algorithm is implemented for matrix inversion, which has a significant effect on computational complexity. For other ordinary matrix multiplications, we still use the standard method. However, it is also possible to extend the Strassen algorithm to employ it in ordinary matrix multiplication, e.g. multiplication between non-square matrices.

### The first derivative of the maximum likelihood function

In the *t*-th iteration, the first derivative *l* ' (***β***) of the maximum likelihood function has to be updated with the current ***β***^(*t*)^. The *k*-th element of *l* ' (***β***) can be obtained in a distributed manner.

13 $$ {\left.\frac{\partial l}{\partial \beta (k)}\right|}_{\boldsymbol{\beta} ={\boldsymbol{\beta}}^{(t)}} = {\displaystyle \sum_{j=1}^h}{\displaystyle \sum_{i=1}^{n_i}}\left({y}_i^j-P\left({\boldsymbol{x}}_i^j,\ {\boldsymbol{\beta}}^{(t)}\right)\right){x}_i^j(k), $$

where *x*_*i*_^*j*^(*k*) and *β*(*k*) are the *k*-th element of ***x***_*i*_^*j*^ and ***β***^(***t***)^, respectively. Equation (13) shows that we can allow each party to separately compute its own part of first derivative based on local data and we add these results [11]. However, such approach will leak the information of ***β***^(***t***)^ at each iteration. Therefore, we need to securely evaluate Eq. (13) without releasing any intermediary ***β***^(***t***)^. As a result, we need to securely evaluate the exponential function *e*^*x*^ in the boolean circuits. In the proposed framework, we use the Taylor series to approximate the evaluation of *e*^*x*^, such that we only need to handle multiplication and addition operations.

14 $$ {e}^x={\displaystyle \sum_{i=1}^{\infty }}\frac{x^n}{n!}=1+x+\frac{x^2}{2!} + \frac{x^3}{3!}+\frac{x^4}{4!}+\dots $$

To simplify computation and avoid overflow, we set a filter to bound the exponential within the interval between −5 and 5. When the exponentials are greater than 5 or smaller than −5, the evaluation results of the logit function (i.e., 1/(1 + *e*^*x*^)) would be smaller than 6.7 × 10^−2^ according to Eq. (1). Thus, we will not lose much accuracy by using this bound.

Simulations in MATLAB shows that the Taylor series could achieve an approximated result with an error less than 10^−2^, when the maximal order for the expansion is set to 15. To reduce the number of multiplications, we transform the Taylor series to a recursive algorithm.

15 $$ {e}^x\approx 1+x\left(1+\frac{x}{2}\left(\dots \left(1+\frac{x}{14}\left(1+\frac{x}{15}\right)\right)\right)\right). $$

We built a look-up table storing the inversion of integers from 1 to 15, to avoid divisions and to speed up the calculation. For the other divisions involved in the logistic function, we treat them as a matrix of size 1.

It is worth mentioning that all the computations in this section are carried out in a customized Boolean circuit, where all the inputs and intermediary information exchange are protected by the OT protocol and the circuits. The only outputs in plaintext are the learned model parameter ***β*** in the proposed SMAC-GLORE.

## Results

In this section, we first describe computational performance evaluations for fundamental operations, including matrix addition, matrix multiplication and matrix inversion. We then describe accuracy evaluations over real datasets with three features, including the Edinburgh dataset, which contains *T wave inversion*, *Sweating* and *Pain in right arm* features, and three genome datasets [50], where the first two features are *ethnicity groups* and the third feature is a SNP. The last column for each dataset is the intercept.

### Computational performance evaluation

We first evaluated the performance of matrix addition, matrix multiplication and matrix inversion under a 2-party setup. We varied the size of matrices from 1 × 1 to 20 × 20 for each party. We simulated both parties on a 64-bit Ubuntu 14.04 platform with an Intel Xeon CPU at 3.10GHz and 256GB RAM, under the 28-bit fixed-point encoding, where both parties were connected by a 1GB network.

**Fig. 3:**
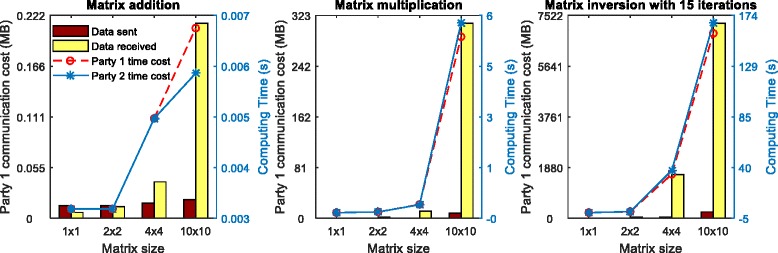
Computational performance in terms of communication costs (i.e., *bar plots*) and circuit computation time (without OT) (i.e., *line plots*) for matrix addition, matrix multiplication and matrix inversion under a 2-party setup

Figure 3 depicts the performance in terms of communication cost (i.e., bar plots) and circuit computation time costs (i.e., line plots) with the same matrix sizes under a 2-party setup for three secure matrix operations. We can see both the computation time and communication costs increase significantly as the matrix sizes increase. This is because the circuit, which implements the functions to be executed, contains more gates as the size of matrix increases. Moreover, secure matrix addition operations have much lower complexity in terms of computation and communication than performing matrix multiplication operations. Among all three secure matrix operations, matrix inversion shows the highest communication and computational demands since it requires several iterations of matrix multiplication and addition operations.

Table 1 shows the computational performance in terms of number of OT and total time cost (including both OT and circuit evaluation costs) for the operations of matrix addition, matrix multiplication and matrix inversion with different matrix sizes in a 2-party setup. For matrix inversion, we measure all the computational performance with 15 iterations. We also included the size of the circuits for different setups in the terms of the total number of gates and the number of AND gates in Table 1, as the evaluation of AND gates is expensive due to the invocations of OT. We can see that the total time increases linearly with the increase of matrix size for matrix addition operations, while the total time increases exponentially for matrix multiplication and matrix inversion operations. This is because the number of AND gates increases exponentially in matrix multiplication and matrix inversion. In the GMW project, where the XOR gates are “free”, only AND gates need secret-sharing evaluations. Therefore, the computing time in matrix multiplication and matrix inversion increases exponentially. Oblivious transfer (OT) is the most computationally expensive part and is the only part which relies on public-key techniques and encryption. During the OT initialization phase, for each wire indexed by *w* in the circuit, GMW generates a secret share *s*_*wi*_ of the input value *v*_*w*_ for each party *P*_*i*_, *i* = 1, …, *h* for a total of *h* parties, and transfers each share to the corresponding party. During the gate-computation procedure, given a wire *w* to be evaluated, all the secret shares *s*_*wi*_ will be re-collected to securely evaluate the gate. Therefore, we can consider the OT initialization as the encryption time and consider the circuit evaluation part or gate- computation part as the decryption time. We also find that in matrix multiplication and inversion, it takes more time for party 2. The bar plots in Fig. 3 show that party 1 receives much more data than it sends out, which is due to the fact that party 2 serves as the sender in OT while party 1 serves as the receiver.

**Table 1:** Computational performance for different matrix sizes in terms of number of gates, OT and total time cost for matrix addition, matrix multiplication and matrix inversion in a 2-party setup

Matrix addition operation						
Matrix size	# of AND gates	# of total gates	OT time (s)		Total time (s)	
			Party 1	Party 2	Party 1	Party 2
1 × 1	27	250	0.346	0.194	0.354	0.202
2 × 2	108	994	0.348	0.194	0.357	0.20
4 × 4	432	2,850	0.343	0.202	0.353	0.212
10 × 10	2,700	24,802	0.369	0.230	0.387	0.247
Matrix multiplication operation						
Matrix size	# of AND gates	# of total gates	OT time (s)		Total time (s)	
			Party 1	Party 2	Party 1	Party 2
1 × 1	4,621	21,594	0.367	0.245	0.384	0.262
2 × 2	37,076	273,034	0.518	0.609	0.577	0.660
4 × 4	580,325	2,707,002	2.135	3.636	2.603	4.060
10 × 10	4,645,300	21,664,002	21.174	50.413	29.646	58.769
Matrix inversion operation (15 iterations)						
Matrix size	# of AND gates	# of total gates	OT time (s)		Total time (s)	
			Party 1	Party 2	Party 1	Party 2
2 × 2	1,030,869	4,872,479	4.864	10.908	6.519	12.472
4 × 4	8,027,793	37,694,207	36.503	85.848	49.619	98.314
6 × 6	26,847,253	125,771,967	121.266	296.780	170.634	349.398
8 × 8	63,345,729	296,412,479	281.653	676.747	405.865	810.214
10 × 10	123,379,701	576,922,463	528.062	1286.500	751.421	1519.897

### Accuracy evaluation

In this section, we perform accuracy evaluation of the proposed framework. As we introduced several approximation schemes (e.g., fixed-point encoding format, Taylor expansion for exponential function, division-free matrix inversion, etc.), the accuracy evaluation intends to measure how the results of the proposed framework differ from those of ordinary methods.

**Fig. 4:**
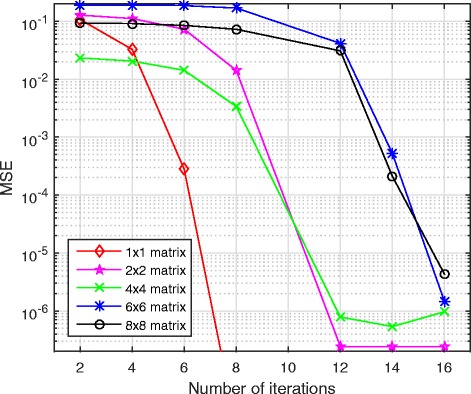
Number of iterations used in secure matrix inversion and MSE for five different matrices

Under the same 2-party settings, we first evaluated the accuracy in terms of mean squared error (MSE) between the secure matrix inversion and ordinary matrix inversion operations. The observed MSEs are mainly due to the use of the fixed-point encoding format and the division-free matrix inversion algorithm. Figure 4 depicts the MSE performance with different matrix inversion iterations using 2 × 2, 4 × 4, 6 × 6, 8 × 8 and 10 × 10 matrices. We find that as the number of iterations increases, the MSEs decrease for all these matrices, and the MSE can get under 10^−5^.

We also evaluated the accuracy of learned coefficients ***β*** in secure distributed logistic regression using different data sets. Our experiments were carried out under two setups: a *local* setting (simulated on a single server, which is the same as the one used in the Computational performance evaluation section) and a *remote* setting (on three different machines). In the *remote* setting, we executed the program on three servers, including the server in the local setting and two other 64-bits Ubuntu servers with Intel Xeon CPUs at 2.40GHz and 96 GB RAM.

In the *local* setting, we simulated both 2-party and 3-party setups. In the *remote* setting with four parties, we hosted the first two parties on a server with 256 GB memory and deployed the other two parties on the remaining two servers. We used the same simulated dataset for all setups, which contains 60 records and 3 binary features. Table 2 compares the results of the learned coefficients, Wald test and two-sample Z test between the proposed SMAC-GLORE and the ordinary logistic regression model. We can see that the proposed SMAC-GLORE framework achieved similar performances as those of ordinary logistic regression, as the outputs are very close, with coefficient differences between 10^−3^ and 10^−2^. Table 3 illustrates the results among 2-party, 3-party and 4-party setups with the same datasets. We can see in Table 3 that all these setups generated exactly the same outcomes. The experimental results demonstrated that the proposed SMAC-GLORE framework enables secure collaboration across multiple parties with high computation precision under different settings.

**Table 2:** Model parameters β learned in SMAC-GLORE and ordinary logistic regression model

*β*	2 parties				Ordinary logistic regression				Two-sample *Z* test	
	Value	Wald test			Value	Wald test			Test statistic	*p*-value
		SE	*Z* value	*p*-value		SE	*Z* value	*p*-value		
*β*_1_	−0.6182	0.7759	−0.7968	0.4256	−0.6274	0.7779	−0.8065	0.4199	0.0084	0.9933
*β*_2_	2.5454	0.8461	3.0084	0.0026	2.5767	0.8511	3.0275	0.0025	−0.0261	0.9792
*β*_3_	1.2246	1.1226	1.0909	0.2753	1.2407	1.1369	1.0913	0.2751	−0.0101	0.9920
*β*_4_	0.6177	0.8283	0.7457	0.4558	0.6198	0.8319	0.7450	0.4562	−0.0018	0.9986

**Table 3:** Model parameters β learned in local 2-party, 3-party scenarios, and remote 4-party scenarios

*β*	2-party				3-party				4-party (remote)			
	Value	Wald test			Value	Wald test			Value	Wald test		
		SE	*Z* value	*p*-value		SE	*Z* value	*p*-value		SE	*Z* value	*p*-value
*β*_1_	−0.6182	0.7759	−0.7968	0.4256	−0.6182	0.7759	−0.7968	0.4256	−0.6182	0.7759	−0.7968	0.4256
*β*_2_	2.5454	0.8461	3.0084	0.0026	2.5454	0.8461	3.0084	0.0026	2.5454	0.8461	3.0084	0.0026
*β*_3_	1.2246	1.1226	1.0909	0.2753	1.2246	1.1226	1.0909	0.2753	1.2246	1.1226	1.0909	0.2753
*β*_4_	0.6177	0.8283	0.7457	0.4558	0.6177	0.8283	0.7457	0.4558	0.6177	0.8283	0.7457	0.4558

In addition, Table 4 shows the performance differences in terms of OT time, circuit computing time and total time for 2-party, 3-party and 4-party setups. We can see that, for different setups, the total number of AND gates are roughly the same. However, the OT and total time costs increase significantly as the number of parties increases. This is because the GWM project requires pairwise OT communication among parties. The circuit computing time is similar between 2-party and 3-party setups under the *local* settings. However, the circuit computing time becomes much larger under the 4-party *remote* setting. This is due to the fact that the servers in the *remote* setting are located under different networks, while the servers in the *local* setting are under the same network. Therefore, the communication cost in remote setting becomes more significant in cross-network setups.

**Table 4:** Computing performances in local 2-party, 3-party scenarios, and remote 4-party scenarios

	2-party		3-party			4-party (remote)			
	Party 1	Party 2	Party 1	Party 2	Party 3	Party 1	Party 2	Party 3	Party 4
OT time (s)	1,785.51	4,021.83	5,075.79	5,192.17	5,168.24	7,225.62	15,927.10	10,051.50	9,929.10
Computing time (s)	601.76	625.59	538.79	659.43	612.51	650.65	1288.07	1011.14	857.72
Total time (s)	2,607.11	4,683.56	5,822.17	6,064.08	5,976.22	8123.55	17,556.40	11,412.60	11,026.5
# of AND gates	355,074,784		355,075,216			355,075,648			

In Table 5, we performed the comparison of models learned from SMAC-GLORE and ordinary logistic regression, based on four real data sets. All the datasets consist of 3 features. Dataset 1 is the Edinburgh dataset and Datasets 2–4 are genome datasets [50]. We can see that our SMAC-GLORE produced very close results to those produced by ordinary logistic regression.

**Table 5:** Differences between models learned from SMAC-GLORE and Ordinary Logistic Regression (LR) for Datasets 1-5

*β*	Dataset 1		Dataset 2		Dataset 3		Dataset 4	
	2-party SMAC-GLORE	Ordinary LR	2-party SMAC-GLORE	Ordinary LR	2-party SMAC-GLORE	Ordinary LR	2-party SMAC-GLORE	Ordinary LR
*β*_1_	1.7632	1.7647	−0.6592	−0.6567	−0.5093	−0.5066	−1.5126	−1.5168
*β*_2_	0.3369	0.3374	0.3174	0.3179	0.5767	0.5777	−0.3516	−0.3488
*β*_3_	1.1885	1.1902	−0.2212	−0.2195	0.4102	0.4138	0.2822	0.2855
*β*_4_	−1.6514	−1.6500	−1.3115	−1.3098	−1.8940	−1.8939	−1.4873	−1.4873

## Limitations and discussion

Our project, which is in part based on the GMW project, developed secure fixed-point algorithms to handle floating number computation and constructed pipelines for securely building a distributed logistic regression model. The experimental results demonstrated the feasibility of the proposed framework, but there are still some limitations.

First, all the variables are represented in a 28-bit fixed-point format, where we allocate 1 bit for the sign (i.e., positive or negative numbers) and 11 bits for fractional part of a floating number. Therefore, the proposed framework cannot handle a number that is larger than 2^16^ or has precision higher than $\frac{1}{2^{11}}$. Although the range and precision of a floating number can be improved by adding more bits in the fixed-point format, it will result in a significant increase of circuit size. Another potential workaround is to replace the fixed-point format with a floating-point format, which will require further investigation. In addition, during fixed-point multiplication, results may end up taking twice the number of bits, where the intermediary product results have 56 bits. In order to maintain a consistent 28-bit fixed-point encoding format, we need to truncate the intermediary product results, which may also result in precision loss. Based on our simulation, we need at least an 80-bit fixed point representation to achieve precision of a single precision floating point encoding format.

Second, to handle secure distributed exponential and logit function evaluations, we implemented a secure Taylor expansion algorithm up to a 15 order and truncated the input range (i.e., −5 to 5). The output precision of both functions can be further improved by increasing the order of expansion and of the truncated input range, but this results in additional computational costs. Similarly, we also developed a secure matrix inversion protocol based on an iterative algorithm [41], which only requires multiplication and addition operations. This secure matrix inversion protocol may also introduce precision loss in the final results.

Third, the current implication of the secure multiplication primitive is partially based on the Strassen algorithm, which requires several levels of block-wise decompositions of the input matrix to achieve maximum performance gain. However, we only perform a one-level decomposition rather than repeating the procedure in the whole multiplication. As matrix multiplication is one of the most time-consuming operations, we may need to fully utilize the Strassen algorithm to improve the performance in our future work.

In addition, based on our experiments, we find that the OT phase contributes the most to the computational time, due to the limitations of the GMW project which uses only a single thread for each pair’s OT procedure. To reduce the time for OT and improve performance, we can resort to parallel computation in the OT procedure. The GMW project was developed for a 32-bit computing environment, which means that the total number of gates cannot exceed 2^31^. Therefore, in our future work, we plan to extend GMW to support a 64-bit address space, in which we can handle larger data sets. Another limitation is that the GMW project needs to preload the entire circuit into memory during the computation, which requires a very large amount of memory for a complex circuit. In our future work, we will investigate the possibility of dynamically generating a part of a circuit that will be required for the next execution to reduce memory consumption.

Although the proposed framework can protect the entire model learning phase, there is still no protection of the final output of the learned model parameter. Differential privacy has emerged as one of the strongest privacy guarantees for statistical data release [51]. Roughly speaking, it ensures (to a pre-defined extent, and quantifiably) that no risk is incurred when data from individual patients are entered in a particular database. It will be useful to integrate an optional component to enable differentially private model learning [52] in our future work.

## Conclusion

In this study, we developed a secret-sharing, circuit-based SMAC-GLORE framework. To overcome the limitation of GMW, which only supports integer operations, we designed a fixed-point encoding format to support floating number arithmetic in SMAC-GLORE. We also implemented several secure matrix-operation primitives and built a pipeline for secure distributed logistic regression calculation. SMAC-GLORE is able to build a shared model without sharing each party’s private data. To the best of the authors’ knowledge, the proposed SMAC-GLORE is the first attempt to enable secret-sharing circuit based secure distributed logistic regression model learning for biomedical research studies. The experimental results show that our framework is reliable and can be used to solve practical problems in secure distributed logistic regression model learning.

## Abbreviations

CSP, computation service provider; CRYSP, crypto service provider; GMW, Goldreich, Micali, and Wigderson; MSE, mean squared error; OT, oblivious transfer; SMC, secure multi-party computation; SMAC-GLORE, secure multi-pArty computation grid logistic regression
